# Puerarin inhibits titanium particle‐induced osteolysis and RANKL‐induced osteoclastogenesis via suppression of the NF‐κB signaling pathway

**DOI:** 10.1111/jcmm.15821

**Published:** 2020-09-07

**Authors:** Wenkai Tang, Long Xiao, Gaoran Ge, Mengdan Zhong, Jie Zhu, Jialin Qin, Chencheng Feng, Wenhao Zhang, Jiaxiang Bai, Xuesong Zhu, Minggang Wei, Dechun Geng, Zhirong Wang

**Affiliations:** ^1^ Department of Orthopedics Zhangjiagang TCM Hospital Affiliated to Nanjing University of Chinese Medicine Zhangjiagang China; ^2^ Department of Orthopaedics The First Affiliated Hospital of Soochow University Suzhou China; ^3^ Center Laboratory Zhangjiagang TCM Hospital Affiliated to Nanjing University of Chinese Medicine Zhangjiagang China; ^4^ Department of Endocrinology Zhangjiagang TCM Hospital Affiliated to Nanjing University of Chinese Medicine Zhangjiagang China; ^5^ Traditional Chinese Medicine The First Affiliated Hospital of Soochow University Suzhou China

**Keywords:** inflammatory, NF‐κB, osteolysis, puerarin

## Abstract

Osteolysis around the prosthesis and subsequent aseptic loosening are the main causes of prosthesis failure. Inflammation due to wear particles and osteoclast activation are the key factors in osteolysis and are also potential targets for the treatment of osteolysis. However, it is not clear whether puerarin can inhibit chronic inflammation and alleviate osteolysis. In this study, we investigated the effect of puerarin on Ti particle‐induced inflammatory osteolysis in vivo in rat femoral models and in vitro in receptor activator of nuclear factor kappa‐B ligand (RANKL)‐induced osteoclast activation models. Our in vivo results showed that puerarin significantly inhibited Ti particle‐induced osteolysis and the expression of matrix metallopeptidase 9 (MMP‐9), nuclear factor of activated T cells 1 (NFATc1), tumour necrosis factor (TNF)‐α and interleukin (IL)‐6. In vitro, puerarin prevented RANKL‐induced osteoclast differentiation, bone resorption and F‐actin ring formation in a concentration‐dependent manner. Furthermore, puerarin decreased the phosphorylation of p65 and prevented p65 moving from the cytoplasm to the nucleus. Puerarin also reduced the expression of osteoclast‐specific factors and inhibited the inflammatory response. In conclusion, our study proves that puerarin can block the NF‐κB signalling pathway to inhibit osteoclast activation and inflammatory processes, which provides a new direction for the treatment of osteolysis‐related diseases.

## 1 INTRODUCTION

Total joint arthroplasty (TJA) has been widely used in clinical treatment of conditions such as femoral head necrosis, trauma, severe osteoarthritis and other diseases.^1^ According to relevant literature reports, approximately 700 000 patients in the United States undergo this surgery each year,^2^ and the demand for surgery is projected to increase rapidly in the next ten years.^3^ However, with increasing time after surgery and the increasing number of patients who have received this treatment, the problem of prosthetic loosening after TJA is becoming increasingly serious and causes great pain and stress for patients and their families.^4^, ^5^ Although developments in science and technology have led to great progress, prosthetic osteolysis remains the most difficult problem.^5^ Wear particles released from the prosthesis, such as titanium particles, play an important role in TJA failure.^6^ Some studies have shown that enoxacin can be safely used to treat particle‐induced peri‐implant osteolysis and other diseases.^7^ Bisphosphonates are considered to directly inhibit osteoclast activity^8^ and enhance bone mass, but they have also been associated with adverse processes in the human jawbone.^9^ Hence, the application of these drugs is limited because of a variety of complications associated with long‐term use.

The reasons for prosthetic loosening are complicated, and the mechanism is not entirely clear. However, it is generally believed that a series of inflammatory reactions caused by wear particles are the most important factors leading to osteolysis around the prosthesis.^10^, ^11^ When wear particles enter the bone around the prosthesis, mononuclear macrophages subsequently phagocytose them and release inflammatory cytokines, such as tumour necrosis factor (TNF)‐α, interleukin (IL)‐1β and IL‐6, causing local chronic inflammation around the prosthesis.^12^ The presence of these factors further stimulates osteoblasts, fibroblasts and bone marrow mesenchymal stem cells to express receptor activator of nuclear factor kappa‐B ligand (RANKL). When combined with RANK on the surface of osteoclast precursor cells, RANKL activates the signalling pathway related to osteoclast differentiation and induces osteoclast formation and bone resorption.^13^ Excessive osteoclast formation disrupts the balance between osteoclasts and osteoblasts in normal bone metabolism.^14^ This ultimately results in osteolysis around the prosthesis and the formation of a fibrous capsule, which leads to loosening of the prosthesis. Therefore, it is necessary to find a drug that can reduce chronic inflammation and inhibit osteoclast function to prevent or treat prosthesis loosening.

Puerarin, which comes from the herb kudzu in traditional Chinese medicine, is an isoflavone compound.^15^ Increasing evidence suggests that puerarin is helpful for hypertension,^16^ Parkinson's disease,^17^ diabetic disease^18^ and tumours.^19^ It was also reported to have anti‐inflammatory and antioxidant effects.^20^, ^21^ A recent study reported that puerarin could promote osteoblast differentiation and new bone formation around implants in the dental implantology field.^22^ The other report provided evidence that puerarin exerted a protective effect in a calvarial osteolysis model.^23^ Another study demonstrated that puerarin stimulates osteoprotegerin (OPG) and affects osteoblastic cells to prevent or retard osteoporosis.^24^ However, it is still unclear whether puerarin could serve as a therapeutic target for the treatment of osteolysis around loosening implants.

**Scheme 1:**
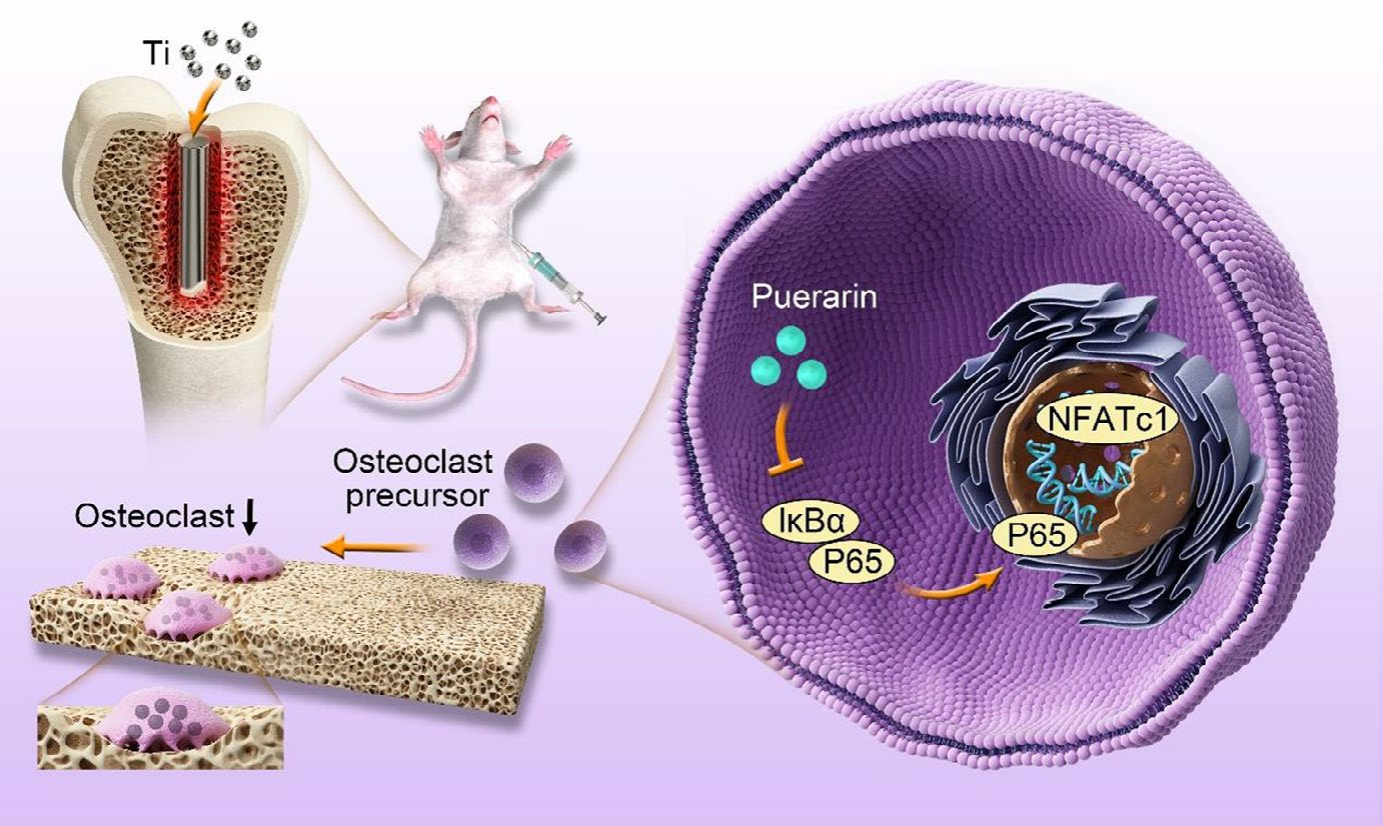
A schematic illustration showing that puerarin has inhibitory effects on osteolysis in vivo and RANKL‐induced osteoclast formation in vitro. Puerarin suppressed the inflammatory response and maintained balance in the bone environment. In addition, puerarin attenuated the upregulation of p65 phosphorylation and the activation of the downstream transcription factor NFATc1. Our results showed that puerarin inhibits osteoclastogenesis by inhibiting the NF‐κB signalling pathway

In this study, we speculated that puerarin has a therapeutic effect on titanium particle‐stimulated inflammatory bone destruction and RANKL‐induced osteoclastogenesis. This hypothesis was tested with rat femur osteolysis models in vivo and in bone marrow‐derived macrophage (BMM)‐ or RAW264.7 cell‐induced osteoclast differentiation models in vitro. We further explored the potential mechanism to verify the inhibitory effect of puerarin on osteoclast activation (Scheme 1).

## 2 MATERIALS AND METHODS

### 2.1 Preparation of Ti particles

Commercial titanium (Ti) powder was purchased from Johnson Matthey Chemical. The mean particle size was 3.32 ± 2.39 mm. More than 90% of the particles were <3.6 mm, 50% were <1.6 mm and 10% were <1.0 mm. Similar to a previous study,^25^ we placed the titanium particles in a suitable sealed container and put them in the oven at 180°C for 6 hours to sterilize them. Then, the titanium particles were transferred to a sterile centrifuge with 75% ethanol for 48 hours. Endotoxin in the titanium particles was detected by a Limulus assay kit (Biowhittaker). We used only endotoxin‐free titanium particles.

### 2.2 Experimental animals and drug treatment

All animal experiments in this study were conducted according to the policy on laboratory animals and were approved by the Institute of Animal Care Committee of Zhangjiagang Traditional Chinese Medicine Hospital (approval number: 2018A013). We established a rat osteolysis model with titanium particle stimulation in the femurs. The experimental animals were male Sprague Dawley rats, which weighed approximately 350 g. Puerarin was purchased from Sigma‐Aldrich. These rats were divided into four groups (n = 5): the (a) control group (surgery only), (b) vehicle group (surgery and 0.1 mL titanium particle suspension in the medullary cavity), (c) low‐dose puerarin group (based on the vehicle group, 15.4 mg/kg of puerarin was injected intraperitoneally every day), and (d) high‐dose puerarin group (based on the vehicle group, 30.8 mg/kg of puerarin was injected intraperitoneally every day). We administered an equal amount of water instead of any medical treatment in the control group and vehicle group. In the first and second weeks, we provided water. From the third week after establishing the model, we performed drug interventions. Four weeks after drug treatment, the rats were sacrificed, and the bilateral femurs were harvested for further study.

### 2.3 Micro‐CT scanning

The right femurs were fixed with 10% formaldehyde for at least 24 hours and then analysed using high‐resolution micro‐CT (SkyScan 1176; SkyScan, Knotich, Belgium). Before scanning, we removed the Ti rods to avoid effects caused by the metal. The femoral scan parameter was set at an equidistant resolution of 9 μm, and the X‐ray energy was set at 80 kV and 100 μA. We used the software provided by the manufacturer to reconstruct the images. For further quantitative analysis, we selected a square region of interest around the titanium rod. Micro‐CT could use 3D reconstruction for measurements, such as bone mineral density (BMD, g/cm^3^), bone volume/tissue volume (BV/TV, %) and bone trabecular thickness (Tb.Th, mm).

### 2.4 Histological and immunofluorescence analysis

After micro‐CT, the harvested rat femurs were decalcified in 10% ethylenediaminetetraacetic acid (EDTA) for 4 weeks. We clipped the length of the femurs to reserve the femoral condyle, which was then embedded in paraffin. Haematoxylin and eosin (H&E) staining was performed. All these samples were cut at a thickness of 5 μm. The stained sections were photographed under an Olympus microscope.

Immunofluorescence (IF) staining of matrix metallopeptidase 9 (MMP‐9), nuclear factor of activated T cells 1 (NFATc1), p65, TNF‐α and IL‐6 was also performed. After dewaxing, antigen repair and sealing, the femur specimens were incubated with primary antibodies (1:1000) at 4°C overnight. Then, the specimens were washed and incubated with secondary antibodies (1:1000) to bind to the primary antibodies for 60 minutes. DAPI (Yeasen Biotech Co., Ltd.) was applied for nuclear staining. Finally, the specimens were coated with resin, and a microscope was used to observe positive cells.

### 2.5 Cell culture and osteoclast differentiation

RAW264.7 cells and BMMs were used in vitro. As previously described,^26^ BMMs were isolated from approximately 6‐week‐old male C57/BL6 mice. BMMs were cultured with Eagle's minimum essential medium, alpha modification (α‐MEM, GE Healthcare Life Science), whereas RAW264.7 cells were cultured with DMEM (GE Healthcare Life Science). The cells were treated with medium containing 10% FBS (Gibco), 50 ng/mL macrophage colony‐stimulating factor (M‐CSF, R&D Systems) and 100 U/mL penicillin‐streptomycin‐amphotericin B (New Cell Molecular) for 48 hours in a 37°C and 5% CO_2_ incubator. Then, the liquid was discarded, and adherent cells were collected. The cells were subcultured at approximately 80% confluence. The cells were stimulated with 50 ng/mL RANKL (R&D Systems) and treated with 0, 1, 10 or 100 μmol/L puerarin dissolved in PBS (Gibco). The medium was changed every other day.

### 2.6 Cell viability assay

To detect the cytotoxic effect of puerarin on BMMs, a cell counting kit‐8 (CCK‐8) viability assay (Beyotime) was performed. BMMs (8 × 10^3 ^cells/well) were plated in 96‐well plates. The BMMs were cultured in induction medium for 24 hours and then treated with various concentrations of puerarin (0, 1, 10, 50,100 or 500 μmol/L) for 1, 3 or 5 days. Ten microlitres of CCK‐8 buffer was added to each well. After incubation at 37°C and 5% CO_2_ for 1 hour, absorbance was measured using a microplate reader (BioTek) at a wavelength of 450 nm.

### 2.7 Pit formation assay

A pit formation assay was performed to determine the effect of puerarin on osteoclast function. BMMs were reseeded in a 24‐well collagen‐coated plate at a density of 3 × 104 cells per well. Next, the cells were treated with puerarin (0, 1, 10, 100 μmol/L) and RANKL (50 ng/mL). After 3 days, we used an inverted microscope to record images of bone absorption areas, which were quantified through ImageJ software.

### 2.8 F‐actin ring immunofluorescence assay

To perform fluorescence staining of the F‐actin ring, various concentrations of puerarin (0, 1, 10 or 100) were added to a 24‐well plate with M‐CSF (50 ng/mL) and RANKL (50 ng/mL) for 7 days. Mature osteoclasts were fixed with 4% paraformaldehyde for 20 minutes and then permeabilized with Triton X‐100 for 10 minutes. The cells were stained with Molecular Probes Alexa Fluor 488 Phalloidin (Cell Signaling Technology) and Alexa Fluor 555 to reveal the cytoskeleton. After the cells were stained with DAPI for 10 minutes, a fluorescence microscope was used for detection.

### 2.9 Quantitative RT‐PCR analysis

Bone marrow‐derived macrophages were isolated from the mouse femur and cultured in a 6‐well plate containing M‐CSF (50 ng/mL) and RANKL (50 ng/mL). The BMMs were administered different doses of puerarin (0, 1, 10, 100 μmol/L) for 5 days. Total RNA was extracted using TRIzol reagent (Ambion). Then, reverse transcription was performed to convert RNA to cDNA. Real‐time PCR was performed with SYBR Green PCR MasterMix. The parameters for PCR were 94°C for 10 minutes, followed by 40 cycles of 95°C for 15 seconds and 60°C for 60 seconds. The sequences for the forward (F) and reverse (R) primers used in this study were as follows:

NFATc1 (forward: 5′‐CAACGCCCTGACCACCGATAG‐3′, reverse: 5′‐GGCTGCCTTCCGTCTCATAGT‐3′),
MMP9 (forward: 5′‐CGTGTCTGGAGATTCGACTTGA‐3′, reverse: 5′‐TTGGAAACTCACACGCCAGA‐3′),
CTSK (forward: 5′‐GGGAGAAAAACCTGAAGC‐3′, reverse: 5′‐ATTCTGGGGACTCAGAGC‐3′), and
GAPDH (forward: 5′‐GGTGAAGGTCGGTGTGAACG‐3′, reverse: 5′‐CTCGCTCCTGGAAGATGGTG‐3′).

### 2.10 Western blot analysis

Total proteins were isolated from RAW264.7 cells induced with RANKL (50 ng/mL) in a 6‐well plate. After treatment with different concentrations of puerarin for the indicated times, protein samples were separated in an electrophoresis apparatus and then transferred to PVDF membranes (Millipore). Next, the membranes were blocked and incubated with rabbit primary antibodies at 4°C overnight. The antibodies used were as follows: ATPase (1:1000, Ab2819), Alpha V (1:1000, Ab179475), Cathepsin K (CTSK) (1:1000, Ab19027), MMP‐9 (1:1000, Ab38898), NFATc1 (1:1000, Ab25916), TNF‐α (1:1000, Ab1793), IL‐6 (1:1000, Ab208113), IκB‐α (1:1000, Ab32518), NF‐κB (1:1000, Ab16502), p‐IκB‐α (1:1000, 2859) and p‐NF‐κB (1:1000, 3031, Cell Signaling Technology). After washing with TBST (CWBiotech) for 15 minutes, the membranes were incubated with secondary antibodies for 2 hours. Finally, the protein was measured with a chemiluminescent HRP substrate (Millipore Corporation).

#### 2.10.1 Statistical analysis

SPSS 19.0 software was used for statistical analysis in this experiment. All the experimental data are expressed as the mean ± standard deviation (M ± SD). Each experiment was independently repeated at least three times. One‐way analysis of variance (ANOVA) was used for statistical comparisons among more than two groups. The post hoc Newman‐Keuls test was suitable for comparisons of two groups. When the *P* value was <.05, the difference was considered statistically significant.

## 3 RESULTS

### 3.1 Puerarin prevented bone loss and inhibited Ti particle‐induced osteolysis

**Figure 1:**
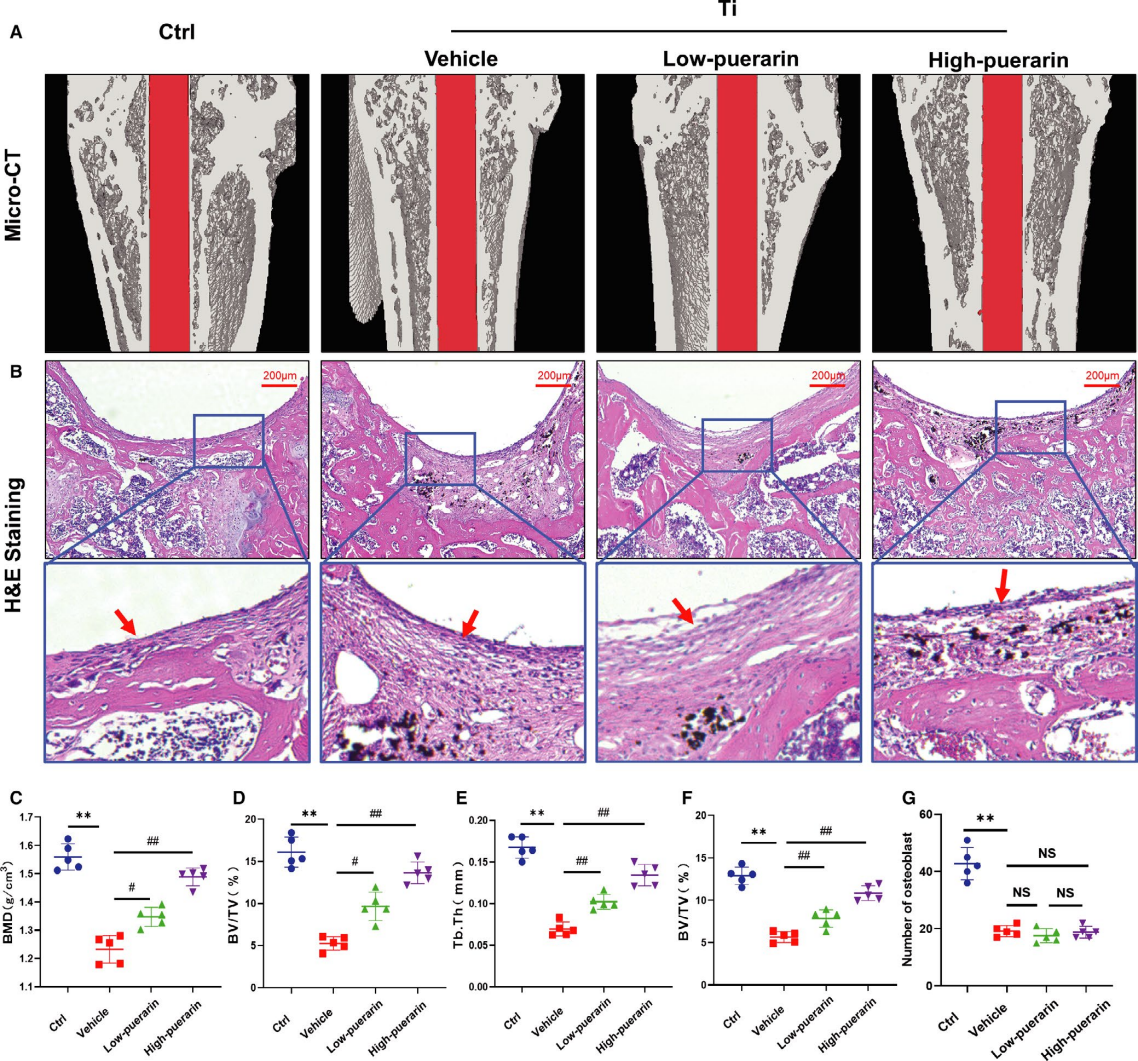
Puerarin attenuates Ti particle‐induced rat femur osteolysis in vivo. (A) Representative micro‐CT 3D reconstruction. (B) Representative paraffinized sections following H&E staining. Red arrows represent inflammatory fibrous tissue. (C) The BMD within the region of interest (ROI) was calculated. (D) BV/TV. (E) Tb.Th. (F) BV/TV. (G) The number of osteoblasts around Ti implants. n = 5; scale bar = 200 μm; **P < .01, #*P* < .05, ##*P* < .01, NS: Not statistically significant, * vs the Ctrl group, # vs the vehicle group

The micro‐CT results showed that the rat femurs treated with Ti particles exhibited osteopenia and bone trabecular disturbance. The destruction of the femoral surface was relieved after treatment with puerarin, which suggested that puerarin reduced bone destruction and maintained bone integrity (Figure 1A). The parameters in the vehicle group decreased significantly compared with those in the control group (BMD: 1.23 g/cm^3^ ± 0.049 g/cm^3^ vs 1.56 g/cm^3^ ± 0.046 g/cm^3^, BV/TV: 5.26% ± 0.81% vs 16.08% ± 1.79%, Tb.th: 0.07 mm ± 0.008 mm vs 0.17 mm ± 0.012 mm, respectively). After treatment with low and high concentrations of puerarin, all these parameters increased compared with those of the vehicle group (*P* < .05) (Figure 1C‐E). H&E staining of the surrounding tissues clearly showed a fibrous capsule between the bone matrix and the Ti rod in the titanium particle groups, indicating that a significant inflammatory reaction was induced by Ti particles. In contrast, a more complete bone structure was observed in the control and high‐dose puerarin groups than in the vehicle and low‐dose puerarin groups. In addition, puerarin treatment significantly increased the BV/TV value in a dose‐dependent manner (Figure 1B,F). This result was consistent with the micro‐CT results, which further suggested that puerarin could reduce local inflammation and inhibit osteoclastogenesis.

**Figure 2:**
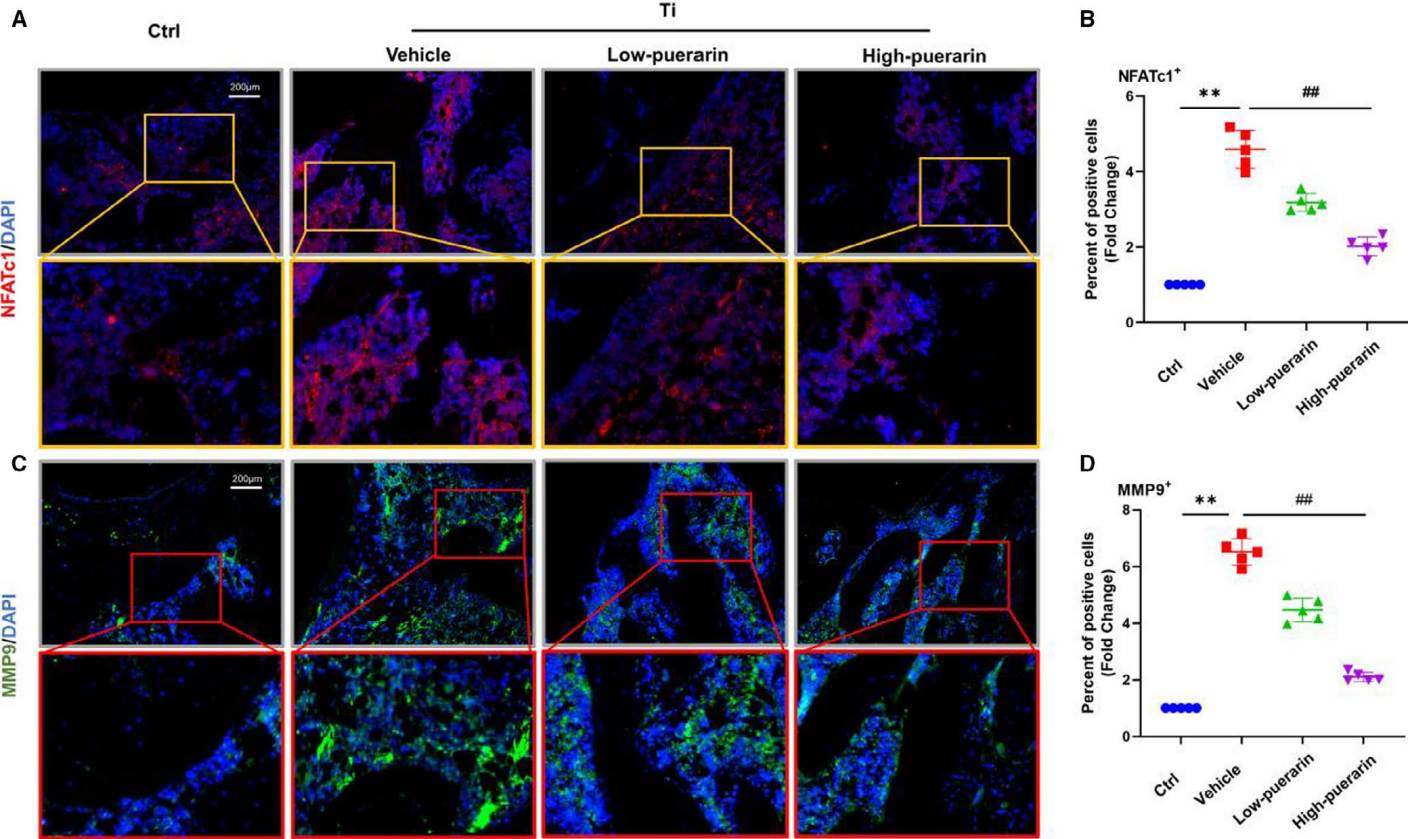
Puerarin inhibits osteoclastogenesis in vivo. (A and C) Representative images showing cells stained for the osteoclastogenesis genes NFATc1 (red) and MMP9 (green) and nuclei (blue) observed by fluorescence microscopy. (B and D) Percentage of NFATc1‐ and MMP9‐positive cells. n = 5; scale bar = 200 μm; **P < .01, ##*P* < .01, * vs the Ctrl group, # vs the vehicle group

NFATc1 and MMP‐9 are important markers that promote osteoclast differentiation. To explore the inhibitory effects of puerarin on osteoclastogenesis, we performed immunofluorescence staining of NFATc1 and MMP‐9. As shown in Figure 2A‐D, positive cells were observed around the trabeculae in the control group. The fluorescence intensity was enhanced in the vehicle group, which represents an increase in the number of positive cells (*P* < .05). In contrast, few positive cells were observed in both puerarin treatment groups (*P* < .05). In addition, no adverse events were recorded in animal experiments. H&E staining of the liver and kidney showed that puerarin treatment had no significant side effect (Figure S1).

### 3.2 Puerarin inhibited RANKL‐induced osteoclastogenesis

**Figure 3:**
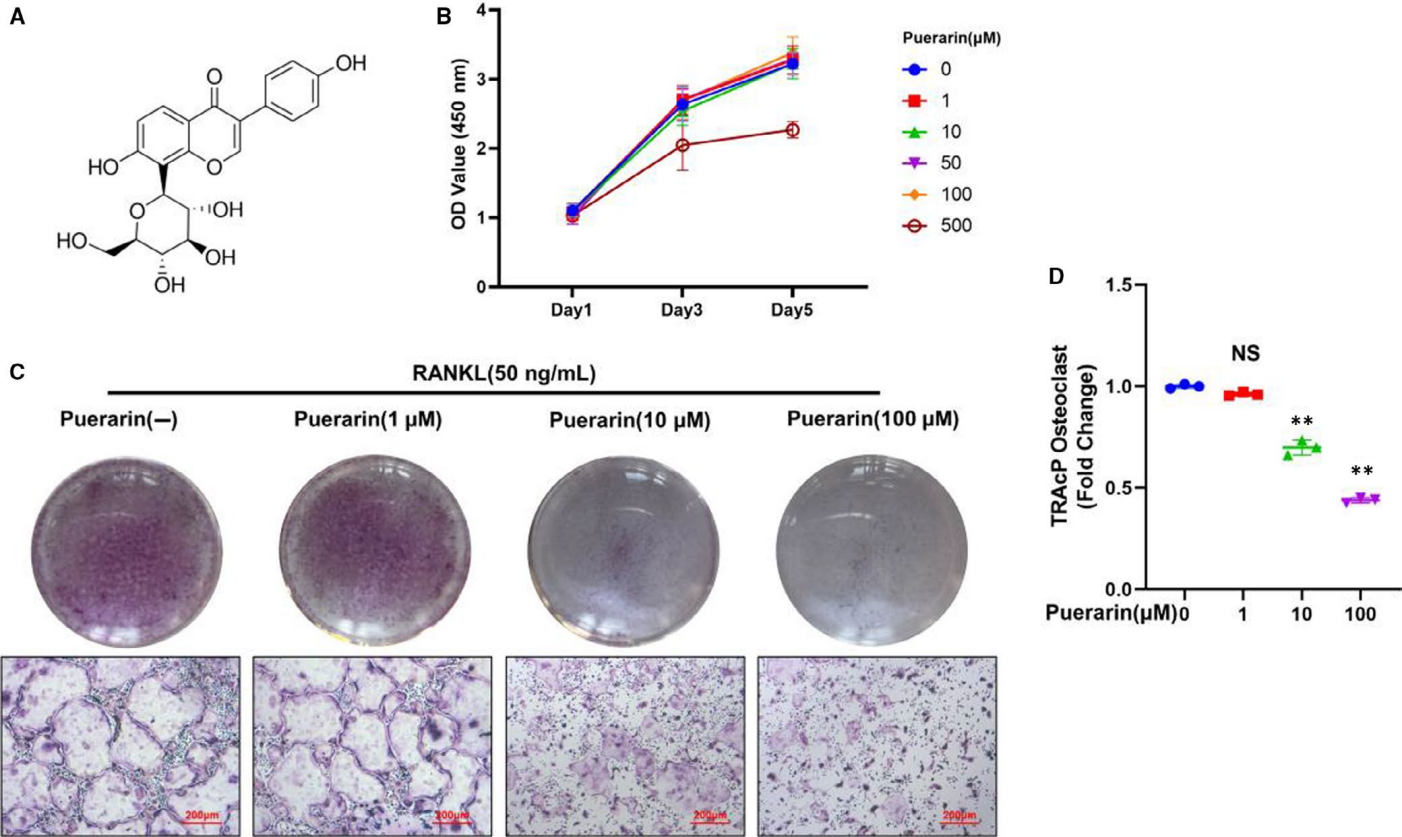
Puerarin inhibited RANKL‐induced osteoclast formation in vitro. (A) Structure of puerarin. (B) A CCK‐8 assay was conducted to evaluate cell viability. (C) Representative images of TRAcP staining. (D) The percentage of TRAcP osteoclasts in each group was quantified. n = 3; scale bar = 200 μm; NS: Not statistically significant, **P < .01, * vs the 0 μmol/L group

The CCK‐8 cell viability assay was used to exclude the possibility that the effects of puerarin on osteoclast formation were due to cytotoxicity. The results showed that cell viability was unaffected by treatment with puerarin at levels below 100 μmol/L for 1, 3 and 5 days (Figure 3A,B).

Because puerarin decreased the number of NFATc1‐ and MMP‐9‐positive cells in vivo, we further speculated that puerarin may prevent wear particle‐induced bone destruction by inhibiting osteoclastogenesis. BMMs were cultivated with M‐CSF (50 ng/mL) and RANKL (50 ng/mL) together with or without various concentrations of puerarin (1, 10, 100 μmol/L) for 5 days. As shown in Figure 3C,D, large claret‐coloured cells were observed in the RANKL groups. However, puerarin reduced the proportion of TRAP‐positive cells following RANKL stimulation in a dose‐dependent manner.

### 3.3 Puerarin influenced osteoclast bone resorption and F‐actin ring formation

**Figure 4:**
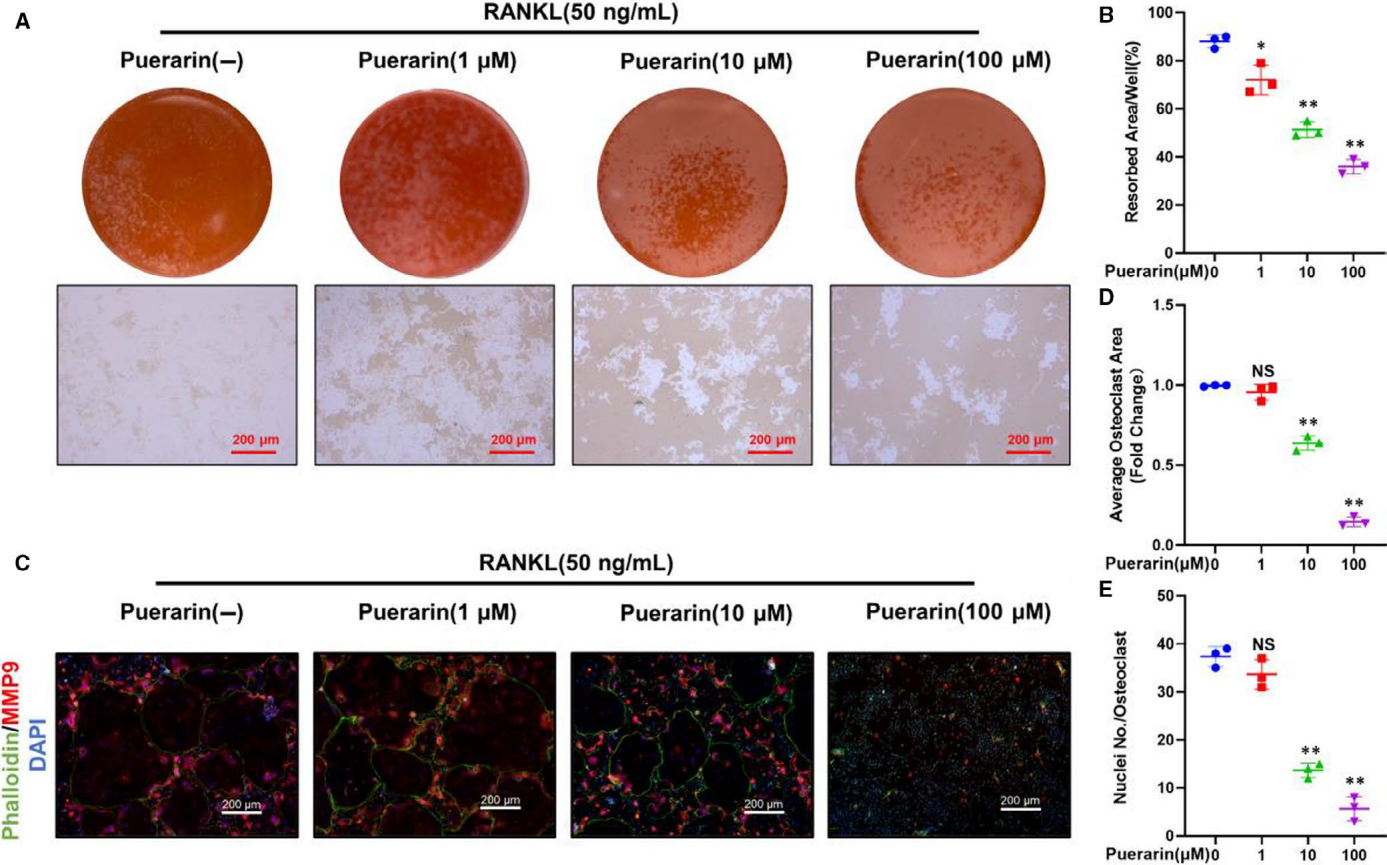
Puerarin influenced osteoclast bone resorption and F‐actin ring formation in vitro. (A) Representative images of bone resorption. (B) Quantification of resorption area/well. (C) Representative images of cells stained with phalloidin, MMP9 and DAPI. (D and E) Quantification of the average osteoclast area and number of nuclei/osteoclast. n = 3; scale bar = 200 μm; NS: Not statistically significant, *P < .05, **P < .01, * vs the 0 μmol/L group

To test whether puerarin could attenuate osteoclastic bone resorption, BMMs were treated in the presence or absence of various concentrations of puerarin. As expected, in the RANKL group, large bone resorption pits were observed on the surface, and the area of resorption reached 88% ± 2.65%. By comparison, the resorption areas dropped to approximately 72% ± 6.25%, 51% ± 3.21% and 36% ± 3% after treatment with 1, 10 and 100 μmol/L puerarin, respectively (Figure 4A,B). The data showed that osteoclast function was inhibited by puerarin in vitro.

It is vital to keep F‐actin well polarized during osteoclast formation. Thus, we decided to test the effects of puerarin on F‐actin ring formation. A typical and intact ringed structure of F‐actin induced by RANKL was observed using immunofluorescence. However, after treatment with puerarin, the size of the F‐actin rings was visibly decreased. In addition, the number and colour of rings were also reduced. These results together suggested that puerarin could inhibit both the generation of F‐actin rings and osteoclastic function (Figure 4C,D).

### 3.4 Puerarin inhibited the expression of osteoclast‐related proteins and genes

**Figure 5:**
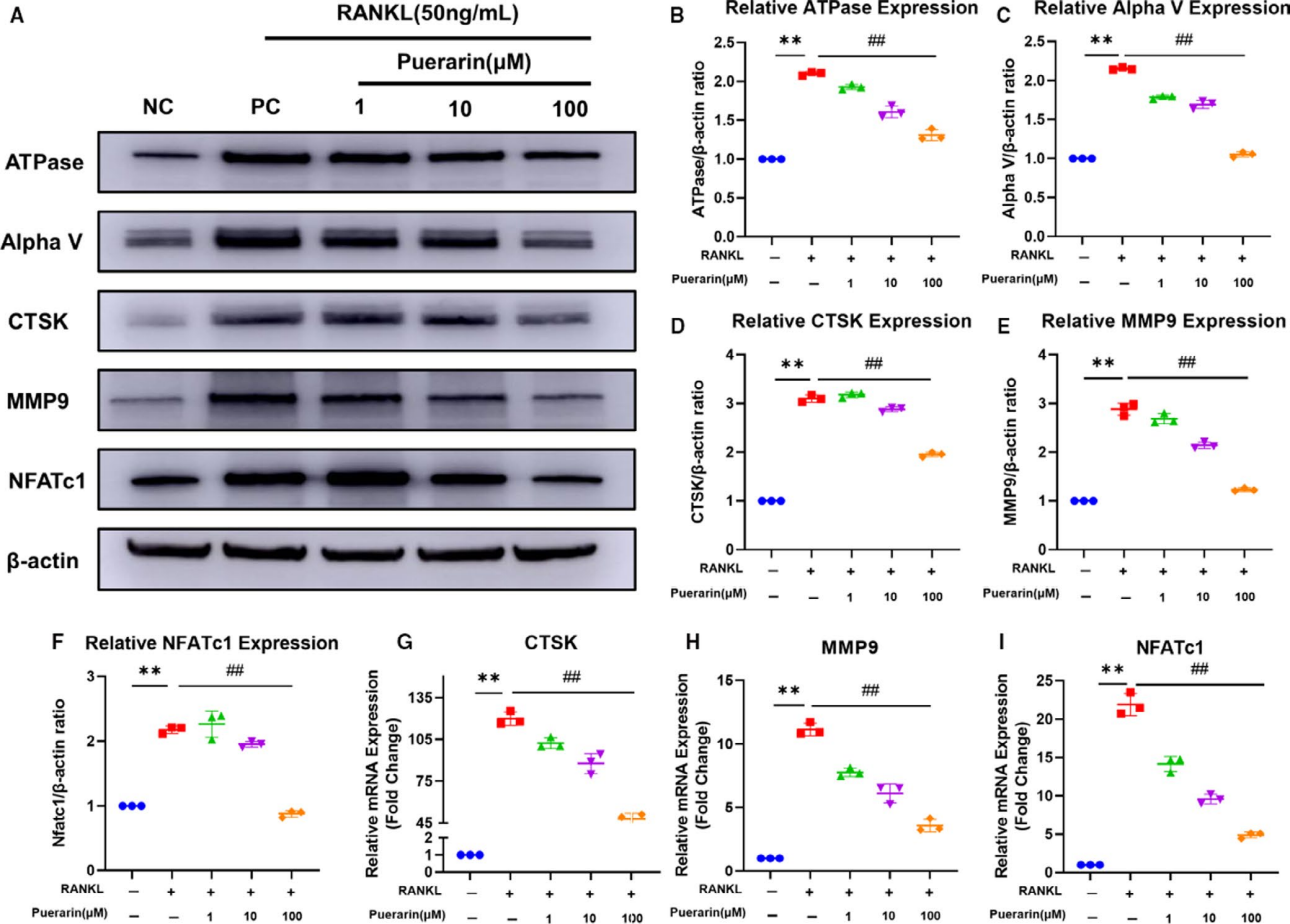
Puerarin inhibited the expression of osteoclast‐related genes and proteins. (A) Cell lysate was subjected to Western blotting with antibodies against the osteoclast‐related proteins ATPase, Alpha V, CTSK, NFATc1 and MMP9. (B‐F) Quantification of these proteins. (G‐I) Quantification of mRNA expression of CTSK, MMP9 and NFATc. n = 3; **P < .01, ##*P* < .01, * vs the NC group, # vs the PC group

Osteoclast differentiation and activation are associated with RANKL.^27^ Western blotting and real‐time PCR were applied to assess whether puerarin suppressed RANKL‐stimulated osteoclast‐related protein and gene expression. BMMs or RAW264.7 cells were treated with induction medium containing RANKL and cultured with various concentrations of puerarin (1, 10, 100 µmol/L). The Western blot (WB) results demonstrated that the expression of ATPase, Alpha V, CTSK, NFATc1 and MMP‐9 was markedly downregulated by puerarin treatment (*P* < .05) (Figure 5A‐F). As shown in Figure 5G‐I, the expression of all genes was elevated after RANKL induction. Compared with RANKL, puerarin significantly decreased the mRNA levels in a concentration‐dependent manner. Therefore, these results further confirmed that puerarin could inhibit expression of osteoclast‐specific factors during osteoclast differentiation in vitro.

### 3.5 Puerarin suppressed activation of the NF‐κB signalling pathway during osteoclastogenesis

**Figure 6:**
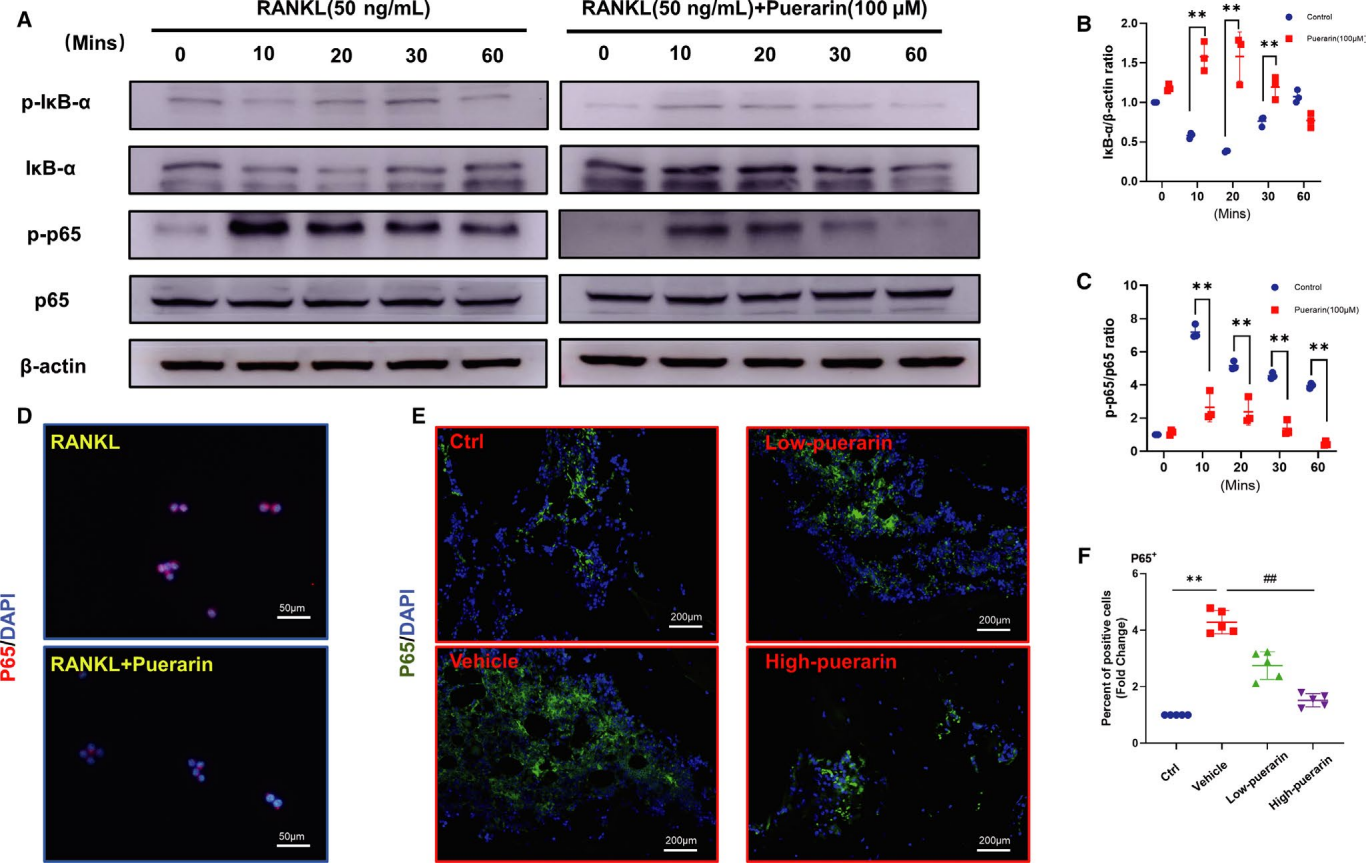
Puerarin suppressed the activation of the NF‐κB signalling pathway during osteoclastogenesis. (A) Cell lysate was subjected to Western blotting with antibodies against phosphor‐IκB‐α, IκB‐α, phosphor‐p65 and p65. (B and C) The ratio of IκB‐α/β‐actin and p‐p65/p65, n = 3, **P < .01, * vs control group. (D) Representative images of p65 in the nucleus, scale bar = 20 μm. (E) Representative images of immunofluorescence staining for p65. (F) Quantification of p65‐positive cells. n = 5; **P < .01, ##*P* < .01, * vs the Ctrl group, # vs the vehicle group

As previous studies have shown, activation of the NF‐κB signalling pathway plays an essential role in RANKL‐induced osteoclastogenesis.^28^ To investigate whether this signalling pathway was affected by puerarin, Western blotting analysis was performed. RAW264.7 macrophages were incubated with or without 100 µmol/L puerarin for 4 hours. Then, both groups were induced with 50 ng/mL RANKL for different times (0, 10, 20, 30, 60 minutes). Our results showed that RANKL obviously stimulated the degradation of IκBα after 20 minutes, and the expression levels then returned to normal. The phosphorylation of p65 was enhanced in the RANKL group. In contrast, an inhibitory effect of puerarin treatment on p65 phosphorylation appeared at 20 minutes (Figure 6A,C).

In addition, the immunofluorescence staining results showed that the expression of p65 was mainly observed in the nucleus after stimulation with RANKL. However, in the puerarin‐treated group, little p65 was transferred from the cytoplasm to the nucleus (Figure 6D). This was further confirmed in immunofluorescence analysis in vivo (Figure 6E,F), with an obvious concentration‐dependent decrease in p65, a pivotal component of the NF‐κB signalling pathway, in response to puerarin. In general, these findings confirmed that puerarin had an inhibitory effect on osteoclast activation and suppressed osteoclastogenesis through the NF‐κB signalling pathway.

### 3.6 Puerarin reduced the expression of proinflammatory cytokines

**Figure 7:**
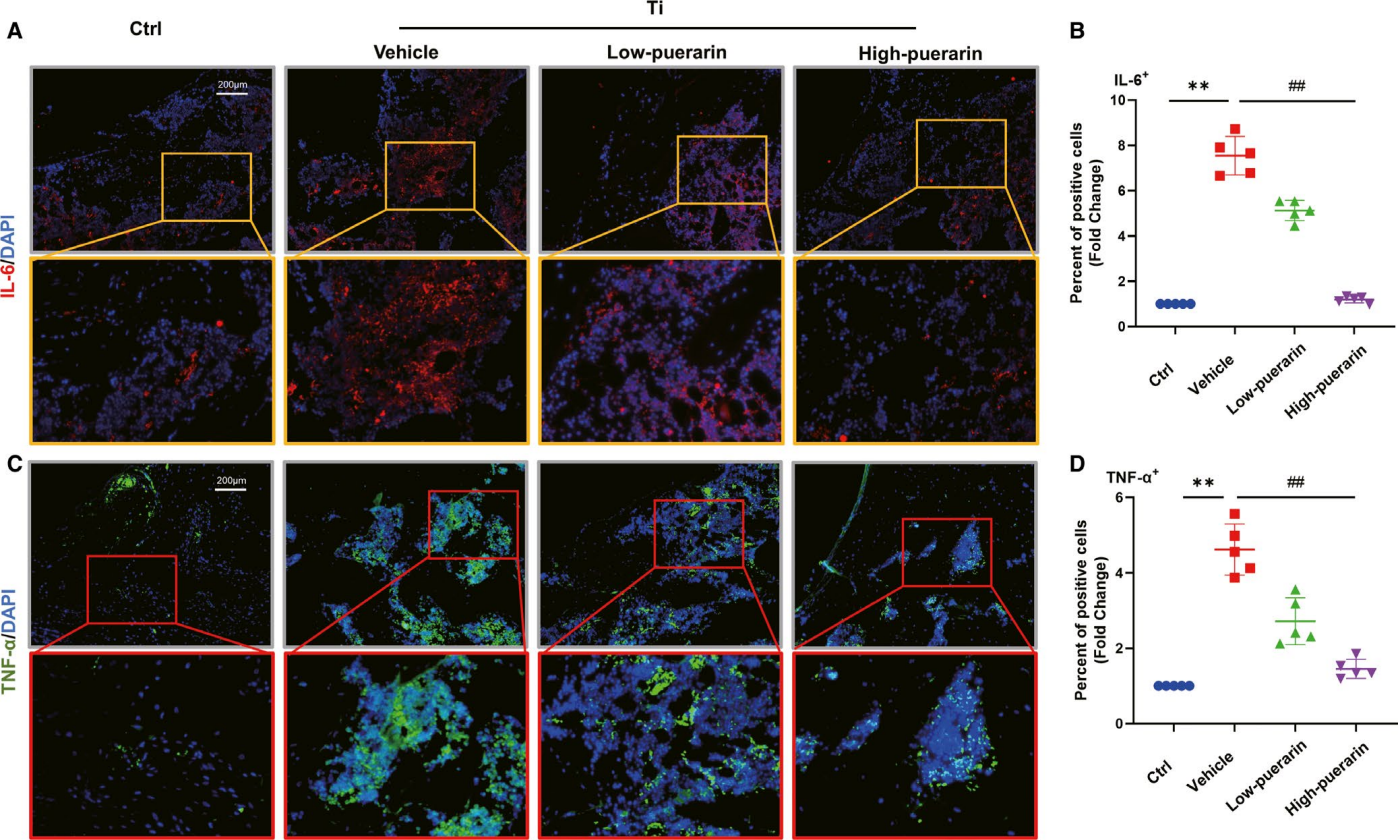
Puerarin reduces the expression of proinflammatory cytokines in vivo. (A and C) Representative images showing cells stained for the inflammatory genes IL‐6 (red) and TNF‐α (green) and nuclei (blue) observed by fluorescence microscopy. (B and D) Percentage of IL‐6‐ and TNF‐α‐positive cells. n = 5; scale bar = 200 μm; **P < .01, ##*P* < .01, * vs the CTRL group, # vs the vehicle group

The inflammatory response is an important component of osteoclast generation.^29^ Immunofluorescence staining in vitro showed that TNF‐α‐ and IL‐6‐positive cells appeared around sites of bone destruction in the control group (Figure 7A‐D). After treatment with Ti particles, the number of positive cells increased significantly, whereas these cytokines were clearly inhibited by puerarin in a dose‐dependent manner. Quantitative analysis indicated that the percentage of TNF‐α‐ and IL‐6‐positive cells in the vehicle group increased approximately fivefold to sevenfold compared with that in the control group (*P* < .05). When different concentrations of puerarin were added to the vehicle group, they reduced TNF‐α and IL‐6 expression to various degrees (*P* < .05). We also observed that puerarin reduced the expression of TNF‐α, IL‐1β and IL‐6 expression in RAW264.7 cells stimulated with RANKL in a dose‐dependent manner (Figure S2). These results indicated that puerarin could inhibit the local inflammatory response.

## 4 DISCUSSION

Aseptic loosening is one of the most common postoperative complications of artificial joint replacement and has also become the most common cause of operation failure. In recent years, great progress has been made in the treatment of osteolytic diseases. Some drugs, such as dracorhodin perchlorate, were found to suppress osteoclast formation and wear particle‐induced osteolysis.^30^, ^31^, ^32^ One of these studies showed that curcumin inhibited RANKL‐mediated osteoclast differentiation and promoted M2‐type macrophage polarization.^33^ Roato I, et al illustrated that T cells promoted osteoclastogenesis and enhanced osteoclast function in an early stage of eriprosthetic osteolysis.^11^ Another study showed that SIRT1 protected osteoblasts against particle‐induced inflammatory responses and apoptosis in aseptic prosthesis loosening.^34^ Therefore, excessive formation of osteoclasts around the prosthesis and overactivity of the osteoclast bone resorption function plays an important role in prosthesis loosening.^35^ In this study, we found that puerarin reduces inflammation and alleviates Ti particle‐induced prosthesis loosening in a rat femur osteolysis model and inhibits osteoclast formation via suppression of the RANKL‐induced NF‐κB pathway. The implant loosening model of the distal femur induced by titanium particles is one of the classical animal models in the study of prosthesis loosening. Our study provides evidence that the increase in osteoclasts caused by Ti particles around the prosthesis causes inflammatory cell infiltration, resulting in an increase in bone absorption, which accelerates bone dissolution around the prosthesis and ultimately leads to a decline in prosthesis stability. This result was consistent with a previous report that inhibiting osteoclast formation and inflammation attenuated wear particle‐induced implant loosening.^36^ All these results proved that puerarin can effectively treat bone loss induced by wear particles in rats.

Osteoclasts, absorbent cells in bone, play an important role in osteolysis.^37^ Studies have verified that a large number of mature osteoclasts are present around osteolysis and cause aseptic loosening.^38^ Our study demonstrated that there were a number of TRAP‐positive cells induced by Ti particles in a rat femur osteolysis model. This result is consistent with a previous study showing that bone‐resorbing osteoclasts increased in the Ti particle group without integrin αvβ3 treatment.^39^ Interestingly, puerarin not only reduced the number of TRAP cells to inhibit osteolysis but also abrogated the expression of TNF‐α and IL‐6 to suppress inflammatory reactions. These inflammatory cytokines are the main factors that cause osteoclast activation.^40^ In conclusion, these results suggested that puerarin reduces wear particle‐induced bone destruction by inhibiting osteoclast activation.

NF‐κB plays a crucial role in osteoclast differentiation, and blocking NF‐κB is a potential strategy for preventing inflammatory osteolysis.^41^ One study showed that mice with p50 and p52 gene knockout developed osteosclerosis during the growth process,^42^ and another demonstrated that NF‐κB signalling pathways are important for inflammation and osteoclast differentiation.^43^ Therefore, activation of the NF‐κB signalling pathway is a necessary process for osteoclast differentiation and maturation.^44^ Previous studies have shown that blocking the NF‐κB pathway reduced the generation of osteoclasts induced by Ti particles.^28^, ^45^ Similarly, in this study, we found that puerarin can inhibit the phosphorylation of IkBa and p65 in RANKL‐mediated osteoclast precursor cells. Furthermore, puerarin also exhibited an inhibitory effect on the nuclear translocation of p65. NFATc1 and MMP‐9 are downstream factors of the NF‐κB pathway and are related to osteoclast activation. The results of this study show that puerarin significantly inhibits the protein expression of NFATc1 and MMP‐9 during osteoclast differentiation. We demonstrate that puerarin inhibits osteoclast formation by suppressing the NF‐κB signalling pathway.

Finally, there are some limitations of our study. First, in this study, we found that puerarin inhibited osteoclast formation to reduce osteolysis. Recently, osteoblasts were observed to play a key role in osteolysis. Some studies have found that puerarin promotes osteoblast‐mediated bone formation.^22^, ^46^ However, it is unclear whether puerarin can improve the inhibition of osteoblast function caused by wear particles in osteolysis. At present, our project team is conducting related work. Second, we adopted a rat femur model with Ti particles in our study, which was confirmed to be similar to osteolysis in the clinic y.^47^ However, the rat femur osteolysis model is not identical to the clinical scenario because the Ti particles are given as a single bolus, and the osteolysis process is studied for a period of only 4 weeks. Thus, a large animal model is required to test the long‐term effectiveness and safety of puerarin interventions.

In summary, this study demonstrated that puerarin effectively alleviated Ti particle‐induced chronic inflammation and osteoclast activation in vivo and disturbed the NF‐κB pathway associated with osteoclast generation and function in vitro. These results further support that puerarin serves as a potential candidate for the treatment of wear particle‐induced osteolysis.

## CONFLICT OF INTEREST

The authors declare no competing financial interest.

## AUTHOR CONTRIBUTIONS

**Wenkai Tang:** Conceptualization (equal); Data curation (equal); Formal analysis (equal); Resources (equal); Software (equal); Writing‐original draft (equal). **Long Xiao:** Data curation (equal); Formal analysis (equal); Methodology (equal); Writing‐original draft (equal). **Gaoran Ge:** Data curation (equal); Formal analysis (equal); Methodology (equal); Resources (equal); Software (equal); Writing‐original draft (equal). **Mengdan Zhong:** Conceptualization (equal); Data curation (equal); Formal analysis (equal); Software (equal); Supervision (equal); Validation (equal); Visualization (equal). **Jie Zhu:** Conceptualization (equal); Investigation (equal); Methodology (equal); Software (equal); Supervision (equal); Visualization (equal). **Jialin Qin:** Conceptualization (equal); Data curation (equal); Resources (equal); Software (equal). **Chengcheng Feng:** Data curation (equal); Formal analysis (equal); Software (equal); Supervision (equal). **Wenhao Zhang:** Conceptualization (equal); Data curation (equal); Resources (equal); Software (equal). **jiaxiang bai:** Data curation (equal); Resources (equal); Software (equal). **Xuesong Zhu:** Project administration (equal); Writing‐review & editing (equal). **Minggang Wei:** Project administration (equal); Writing‐review & editing (equal). **Dechun Geng:** Funding acquisition (equal); Project administration (equal); Writing‐review & editing (equal). **Zhirong Wang:** Conceptualization (equal); Funding acquisition (lead); Project administration (lead); Supervision (equal); Writing‐review & editing (lead).

## Supporting information

FigS1

FigS2

FigS1‐S2

## Data Availability

The date used to support the findings of this study are available from the corresponding author upon request.
