# Supplementary figures and images for: Puerarin inhibits titanium particle‐induced osteolysis and RANKL‐induced osteoclastogenesis via suppression of the NF‐κB signaling pathway
Source: J Cell Mol Med. 2020 Sep 7;24(20):11972–83. doi: 10.1111/jcmm.15821 (PMC7578865; doi:10.1111/jcmm.15821)

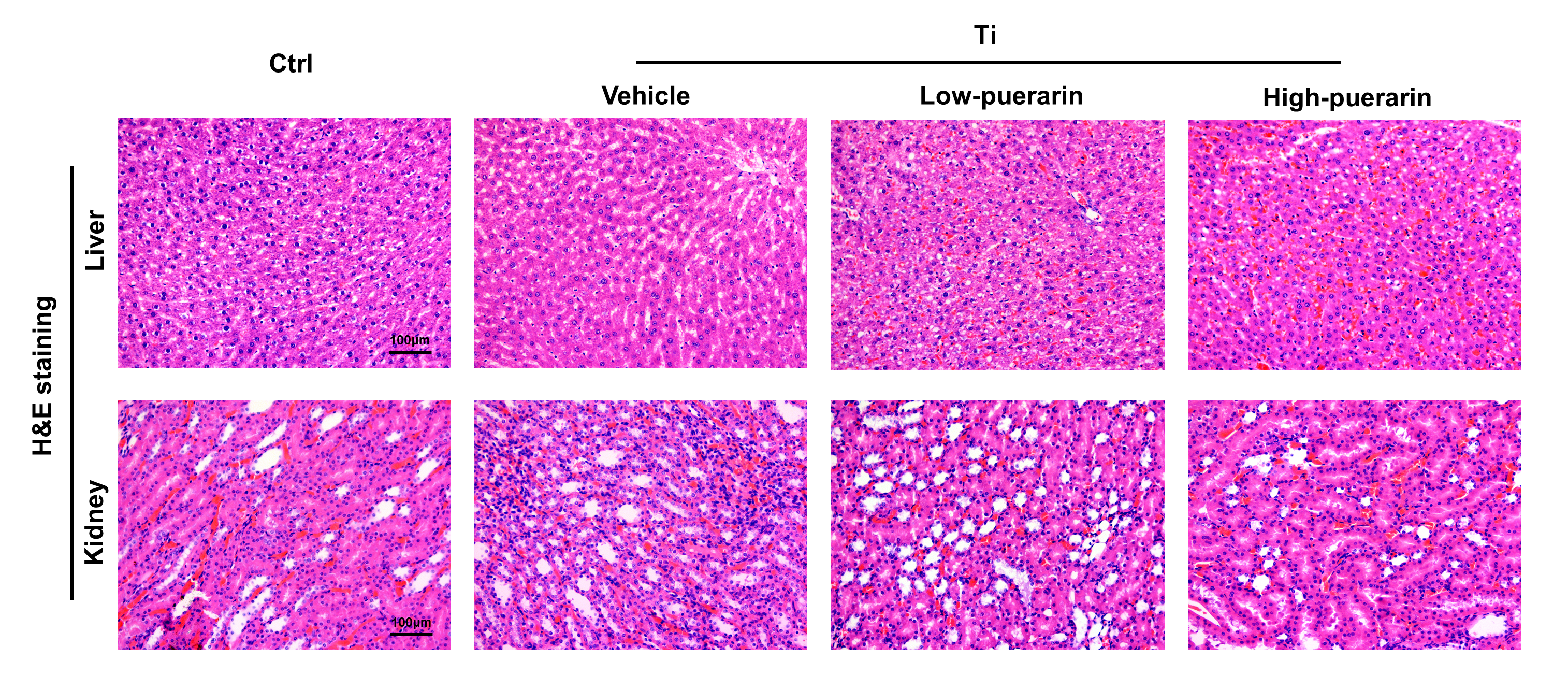

Supplement: Supplementary file 1 — FigS1 [file JCMM-24-11972-s001.TIF]

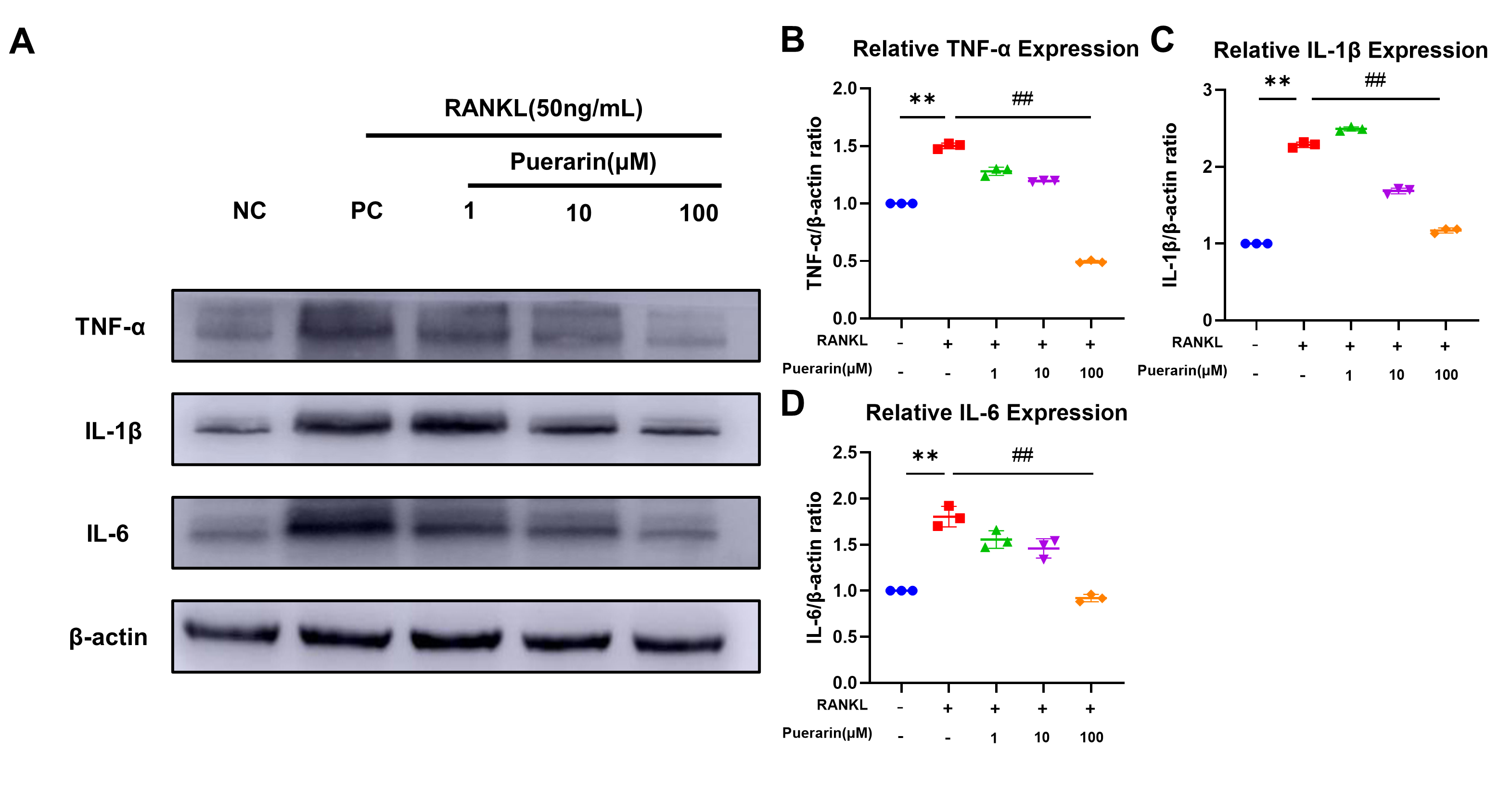

Supplement: Supplementary file 2 — FigS2 [file JCMM-24-11972-s002.TIF]
