# Supplementary material for: Puerarin inhibits titanium particle‐induced osteolysis and RANKL‐induced osteoclastogenesis via suppression of the NF‐κB signaling pathway
Source: J Cell Mol Med. 2020 Sep 7;24(20):11972–83. doi: 10.1111/jcmm.15821 (PMC7578865; doi:10.1111/jcmm.15821)
Supplement: Supplementary file 3 — FigS1‐S2 [file JCMM-24-11972-s003.docx]

## FigureS1


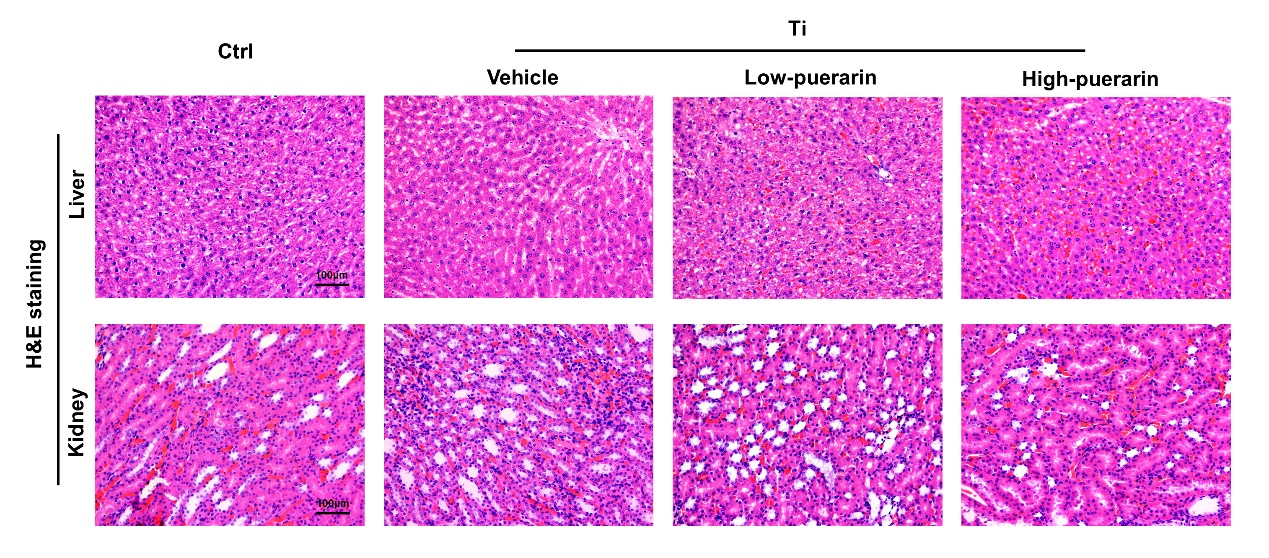


**Figure S****1. H&E staining of the liver and kidney after puerarin treated in vivo**

## FigureS2


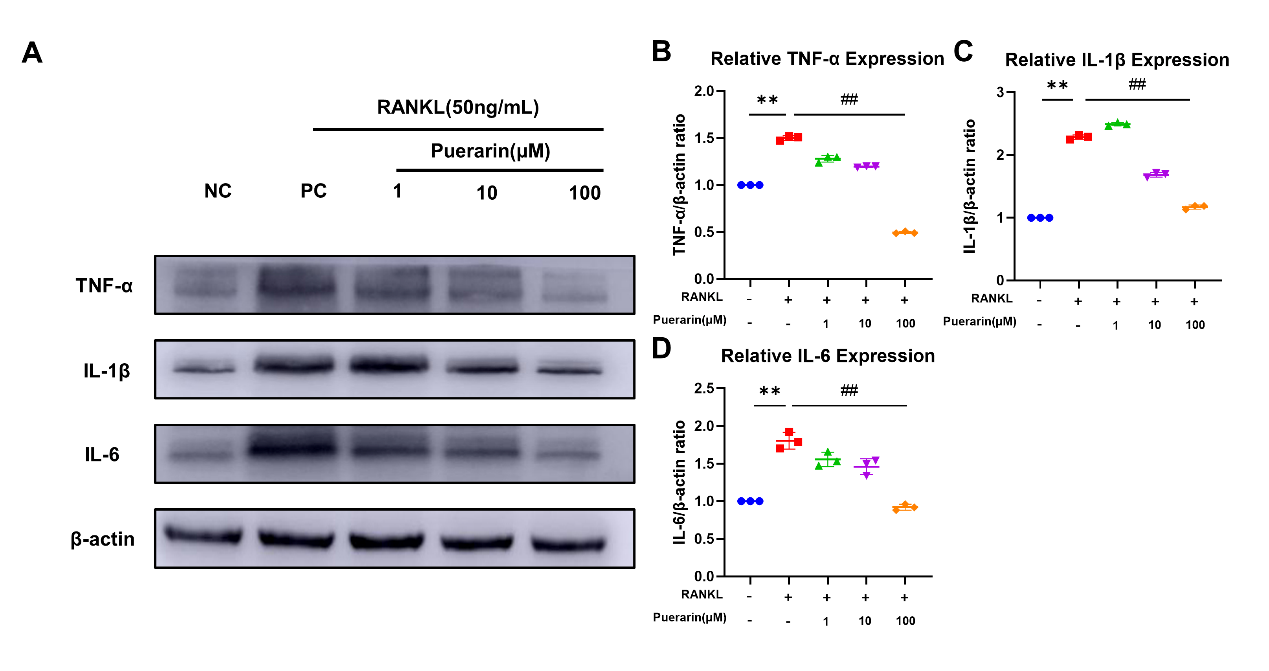


**Figure S2: Puerarin reduces inflammatory cytokine expression in vitro.** a) Cell lysate was subjected to Western blotting with antibodies against TNF-α, IL-1β and IL-6. b-d) The ratio of TNF-α, IL-1β and IL-6. n=3; *p< 0.05, **p < 0.01, #p<0.05, ##p<0.01, * vs. the NC group, # vs. the PC group.
